# Prognostic clinicopathologic factors in carcinoma of unknown primary origin: a study of 106 consecutive cases

**DOI:** 10.18632/oncotarget.16021

**Published:** 2017-03-08

**Authors:** Junjeong Choi, Ji Hae Nahm, Sang Kyum Kim

**Affiliations:** ^1^ College of Pharmacy, Yonsei Institute of Pharmaceutical Sciences, Yonsei University, Incheon, Republic of Korea; ^2^ Department of Pathology, Yonsei University College of Medicine, Seoul, Republic of Korea

**Keywords:** carcinoma of unknown primary origin, CK20, Culine’s prognostic model, prognosis, unfavorable group

## Abstract

A heterogeneous group of cancers for which the site of origin remains occult after detailed investigations is defined as carcinomas of unknown primary origin (CUPs). Because patients with CUP have a dismal prognosis, we have analyzed CUPs to highlight the implication of clinicopathologic factors related with patient survival. A total of 106 consecutive cases of CUP were collected. A two-step strategy of immunohistochemistry to assess CUPs according NCCN Guidelines is used to separate carcinomatous tumors and subtype carcinomas. Median follow up of censored patients was 26 months. Median survival time of whole patients was 13 months (95% confidence interval [CI], 8.43 - 19.1 months), with one, two and five-year survival rate of 53.7%, 35.1%, and 30.5%, respectively. Factors related with shorter overall survival was adenocarcinoma histology (*P*=0.001), increased CA19-9 (*P*=0.003), increased CEA (*P*=0.047), increased LDH (*P*<0.001), CK20 positivity (*P*=0.002), presence of bone metastasis (*P*=0.017), metastasis not confined to the lymph nodes (*P=*0.015), unfavorable clinical group based predefined category (*P*=0.017), and patients with no treatment (*P*<0.001). Multivariable analysis with cox regression model revealed factors related with overall survival; cases belonged to Culine’s poor risk group (HR, 3.88; 95% CI, 1.75-8.64; *P*=0.001) and CK20 positivity (HR, 3.31; 95% CI, 1.42-7.70; *P*=0.005). In conclusion, the CK20 expression profile is a prognostic factor in patients with CUP and initial stratification of patient with Culine’s model may provide a prognostic information in these patients. Assessment of clinical implication of these factors in the context of site specific therapy needs to be evaluated.

## INTRODUCTION

Carcinoma of unknown primary origin (CUP), also known as occult primary tumor is defined as histologically proven metastatic malignant tumors whose primary site cannot be identified by clinical manifestation, radiographic and pathologic examinations [1, 2]. It comprises heterogeneous groups of tumor, clinically and histologically and about 3% to 5% of all newly diagnosed malignant tumors are classified as CUP [2].

Among CUPs, adenocarcinoma comprises the most common histologic type in reported studies [3, 4]. Generally, patients of CUP have poor clinical prognosis with limited life expectancy. Median survival of 3 months in extranodal adenocarcinoma and 8 months in metastatic adenocarcinoma limited to lymph node are expected in population based analysis [4]. In addition, it is rapidly invasive with early dissemination, and shows unpredictable pattern of metastatic spread.

Although the primary site of a CUP remains unknown in 20-50% of patient even after a full diagnostic workup [5], it is known that a subset of patients with CUP shows favorable clinical behavior. Identification of this group seems pivotal in the management of patients. Favorable subsets of patients accounts for about 20% of those with CUPs, and include women with papillary adenocarcinoma of the peritoneal cavity, women with adenocarcinoma involving the axillary lymph nodes, poorly differentiated carcinoma with midline distribution, poorly differentiated neuroendocrine carcinoma, squamous cell carcinoma involving cervical lymph nodes, adenocarcinoma with a colon-cancer profile, men with osteoblastic bone metastases and elevated prostate-specific antigen, isolated inguinal lymphadenopathy with squamous histology, and patients with one, small, or potentially resectable tumor [6].

The best approach of stepwise algorithm with immunohistochemistry (IHC) of biopsied tissue was suggested to provide a specific therapy against the most likely primary site. Although a number of recent studies discussed the use of gene expression-based tests and methylation profile in the setting of CUP, practical value of this approach in the daily clinical practice is still limited [7-10]. Thus, a cost-effective and systemized IHC with identification of clinicopathologic factors related with the patient prognosis are clinically relevant [11, 12]. Most of previous reported publication focused on the usage of appropriate IHC panel for the differential diagnosis. Limited number of publication described general clinical behavior and clinicopathologic factors related with them [13, 14]. In the present study, consecutive cases of CUPs from one institute were analyzed to highlight the implication of clinicopatholoic factors related with patient survival.

## RESULTS

### Patient demographic characteristics and clinical manifestation

**Figure 1:**
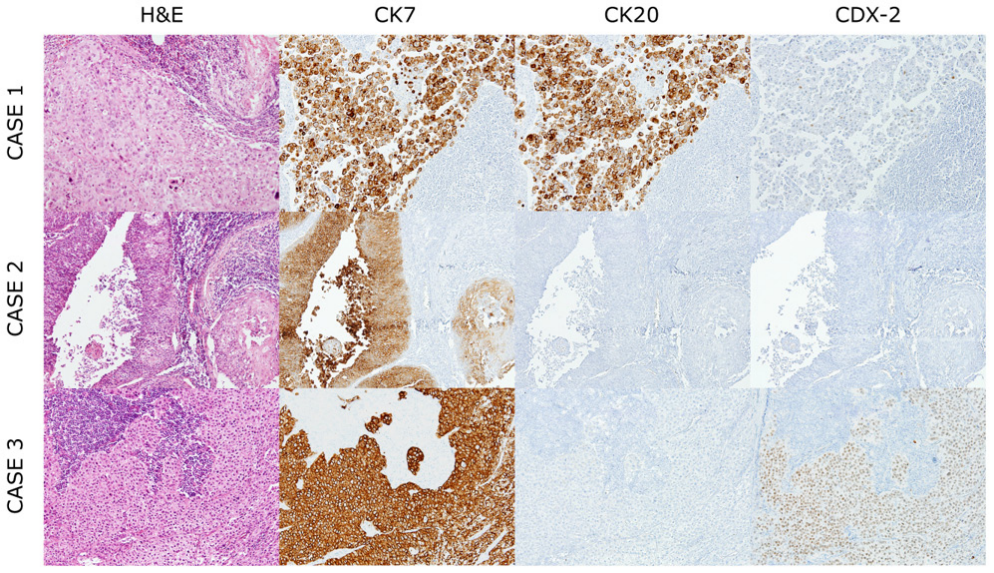
Representative histological features of cancers of unknown primary origin Case1; Poorly differentiated carcinoma, positive for CK7 and CK20 and focal positive for CDX-2. Case2; Squamous cell carcinoma, positive for CK7. Case3; Undifferentiated carcinoma, positive for CK7 and CDX-2 and negative for CK20.

A total of 106 cases from 106 patients were collected from the year of 2000 to 2015. Clinicopathologic characteristics of the patients are presented in Table 1. Median age at time of diagnosis is 59 (range: 25-83) and the number of male patients are sixty nine (65.1%). Most common histologic type was adenocarcinoma (43 cases, 40.6%), followed by squamous cell carcinoma (28 cases, 26.4%), poorly differentiated carcinoma (26 cases, 24.5%) and undifferentiated carcinoma (9 cases, 8.5%). Representative images of H-E stained section are displayed in Figure 1. Cases of poorly differentiated carcinoma showing neuroendocrine differentiation, confirmed by the IHC, were reported in 4 cases.

**Table 1:** Clinicopathologic characteristics of patients with CUP

Variable	Patients N(%)(total=106)	Variable	Patients N(%)(total=106)
**Age(years)**		**Bone Metastasis**	
Median	59(25-83)	Absent	78(73.6)
<50	28(26.4)	Present	28(26.4)
>50	78(73.6)	**Treatment**	
		No treatment	19(17.9)
**Gender**		Operation	40(37.7)
Female	37(34.9)	Chemotherapy	41(38.7)
Male	69(65.1)	Radiation therapy	44(41.5)
**Performance Status(ECOG)**		Concurrent chemo-radiation	16(15.1)
0	13(12.6)		
1	86(81.1)	**CEA**	
2	4(3.8)	Normal	48(45.3)
3	1(0.9)	High	32(30.2)
4	2(1.9)	Not assessed	26((24.5)
**Histology**		**CA19-9**	
Poorly differentiated carcinoma	26(24.5)	Normal	45(42.5)
Adenocarcinoma	43(40.6)	High	26(24.5)
Squamous cell carcinoma	28(26.4)	Not assessed	35(33.0)
Undifferentiated carcinoma	9(8.5)	**CA125**	
**Lactate dehydrogenase(IU/L)**		Normal	16(15.1)
Normal	14(13.2)	High	24((22.6)
High	36(34.0)	Not assessed	66.(62.3)
Not assessed	56(52.8)	**CA15-3**	
**CK7 immunosttinng**		Normal	20(18.9)
Negative	27(25.5)	High	6(5.6)
Positive	60(56.6)	Not assessed	80(75.5)
Not assessed	19(17.9)	**AFP**	
**CK20 Immunostaining**		Normal	48(45.3)
Negative	77(72.6)	High	3(2.8)
Positive	10(9.4)	Not assessed	55(51.9)
Not assessed	19(17.9)	**PIVKA-II**	
**CDX2 Immunostaining**		Normal	18(17.0)
Negative	76(71.7)	Increased	12(11.3)
Positive	11(10.4)	Not assessed	76(71.7)
Not assessed	19(17.9)	**SCCAg**	
**Lung Metastasis**		Normal	20(18.9)
Absent	89(84.0)	Increased	8(7.5)
Present	17(16.0)	Not assessed	78(73.6)
**Liver Metastasis**			
Absent	96(90.6)	**Prognostic group**	
Present	10(9.4)	Favorable	31(29.2)
**Brain Metastasis**		Non-favorable	75(70.8)
Absent	70(66.0)	**Lower GI profile**	
Present	8(7.5)	Lower GI profile	6(5.7)
Symptomatically suspicous	2(1.9)	Non-Lower GI profile	81(76.4)
Not evaluated	26(24.5)	Not assessed	19(17.9)

About 72.6% (77 out of 106) of patients were treated by chemotherapy and radiation concurrently or sequentially. Metastasis to liver, lung, bone and brain was noted in 10 (9.4%), 17 (16.0%), 28 (26.4%) and 10 (9.4%) patients, respectively. Tumor markers at the time of diagnosis were assessed and elevated carcinoembryonic antigen (CEA), cancer antigen (CA) 19-9, CA125, CA15-3, and alpha fetoprotein (AFP) were recorded in 32 (30.2%), 26 (24.5%), 24 (22.6%), 6 (5.6%) and 3 (2.8%) patients, respectively. When CUP patients were categorized into the ‘favorable’ and ‘non-favorable’ groups based on predefined criteria, the number of cases belonging to the favorable group was 31 (29.2%), compatible with the previously known range of about 80%. Among these 19 patients (17.9%) presented as squamous cell carcinoma of the cervical lymph nodes, 4 patients presented as poorly differentiated carcinoma with neuroendocrine characteristics proven by immunohistochemistry, 6 patients with adenocarcinoma with colon-cancer profile and 2 male patients with blastic bone metastases and elevated prostate-specific antigen.

### Univariate analysis of clinicopathologic factors related to the patient’s survival

**Figure 2:**
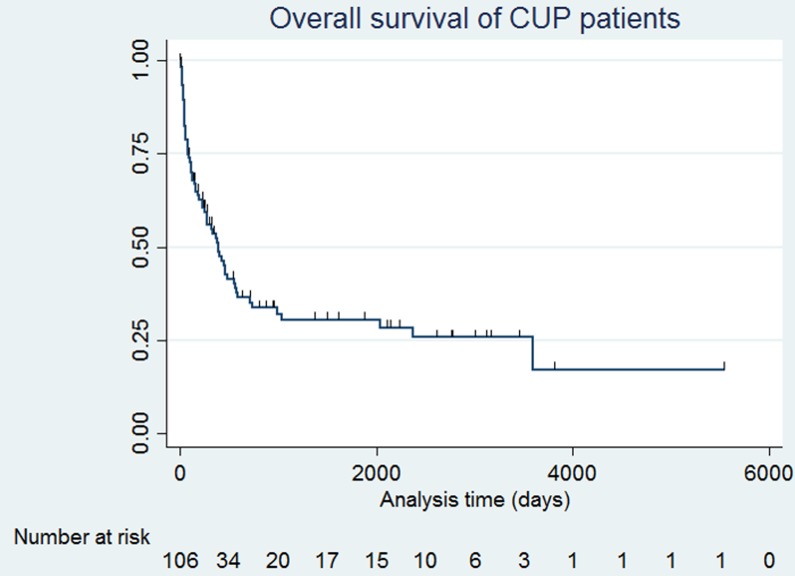
A Kaplan-Meier graph of overall survival of all patients

The overall survival probability for the entire CUP study patients is displayed in Figure 2. A total of 39 cases were censored and death event were 67 cases. Median follow up of censored patients was 26.7 months. Median survival time was 13 months (95% confidence interval [CI], 8.43 - 19.1 months), with one, two and five-year survival rate of 53.7%, 35.1%, and 30.5%,, respectively, which is better than reported (Figure 2).

**Figure 3:**
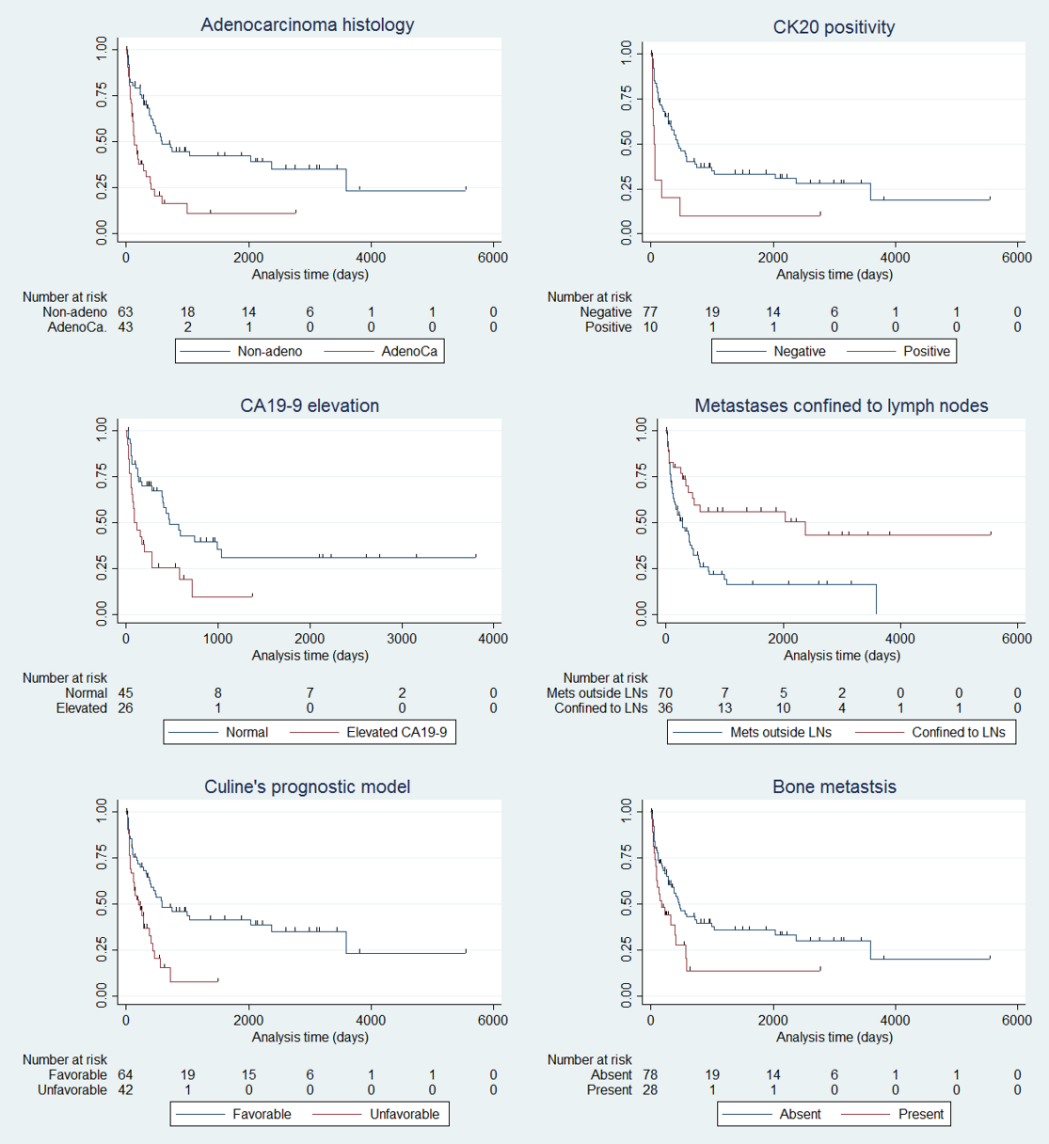
Kaplan-Meier survival estimates of CUP patients according to the clinicopathologic factors Adenocarcinoma histology (*P* = 0.001), increased CA19-9 (*P* = 0.003), patients belonging to poor risk group in Culine’s model (*P* < 0.001), CK20 positivity (*P* = 0.002), metastasis not confined to the lymph nodes (*P* = 0.0015), and presence of bone metastasis (*P* = 0.017) were factors related with unfavorable clinical outcome.

Given the importance of the identification of subgroups with favorable clinical behavior, several clinicopathologic factors were analyzed to identify factors related with overall survival and are summarized in Table 2, Figure 3 and Supplementary Figure 1. Factors related with shorter overall survival was presence of bone metastasis (*P* = 0.017), metastasis not confined to the lymph nodes (*P* = 0.002), patients with no treatment (*P* < 0.001), adenocarcinoma phenotype (*P* < 0.001), increased CA19-9 (*P* = 0.003), increased CEA (*P* = 0.047), patients belonging to poor risk group in Culine’s model (*P* < 0.001) and increased lactate dehydrogenase (LDH, *P* < 0.001). In the female subset, patients with axillary lymph node metastasis showed tendency of better overall survival, although not statistical significant (*P* = 0.057).

**Table 2:** Univariate analysis of clinicopathologic characteristic related with overall survival

Variable	Patients N(%)	Event	Median survival(months)	*P* value
**Age(years)**				
<50	28(26.4)	17	19	0.2238
>50	78(73.6)	50	12	
**Gender**				
Female	37(34.9)	24	12	0.8384
Male	69(65.1)	43	13	
**Performance Status(ECOG)**				
0	13(12.3)	6	78	**0.0014**
1	86(81.1)	57	12	
2	4(3.8)	2	2	
3	1(0.9)	0	0	
4	2(1.9)	2	1	
**Histology**				
Poorly differentiated carcinoma	26(24.5)	15	18	**0.0016**
Adenocarcinoma	43(40.6)	32	4	
Squamous cell carcinoma	28(26.4)	15	24	
Undifferentiated carcinoma	9(8.5)	5	119	
**Lactate dehydrogenase(IU/L)**				
Normal	14(13.2)	4	6	**0.0002**
High	36(34.0)	27	0	
Not assessed	56(52.8)			
**CK7 immunosttinng**				
Negative	27(25.5)	17	24	0.1111
Positive	60(56.6)	42	9	
Not assessed	19(17.9)			
**CK20 Immunostaining**				
Negative	77(72.6)	50	14	**0.002**
Positive	10(9.4)	9	1	
Not assessed	19(17.9)			
**CDX2 Immunostaining**				
Negative	76(71.7)	51	14	0.0418
Positive	11(10.4)	8	1	
Not assessed	19(17.9)			
**Lung Metastasis**				
Absent	89(84.0)	55	2	0.2142
Present	17(16.0)	12	0	
**Liver Metastasis**				
Absent	96(90.6)	60	13	0.4388
Present	10(9.4)	7	7	
**Bone Metastasis**				
Absent	78(73.6)	47	15	**0.0171**
Present	28(26.4)	20	5	
**Brain Metastasis**				
Absent	70(66.0)	38	16	0.6027
Present	8(7.5)	8	24	
Symptomatically suspicous	2(1.9)			
Not evaluated	26(24.5)			
**Treatment**				
No treatment	19(17.9)	10	2	**0.0000**
Operation	40(37.7)	11	6	
Chemotherapy	41(38.7)	13	13	
Radiation therapy	44(41.5)	15	7	
Concurrent chemoradiation	16(15.1)	18	34	
**CEA**				
Normal	48(45.3)	30	14	**0.0468**
High	32(30.2)	23	4	
Not assessed	26(24.5)		0	
**CA19-9**				
Normal	45(42.5)	25	15	**0.0031**
High	26(24.5)	21	2	
Not assessed	35(33.0)		0	
**CA125**				
Normal	16(15.1)	13	12	0.7652
High	24(22.6)	18	4	
Not assessed	66(62.3)		0	
**CA15-3**				
Normal	20(18.9)	13	13	0.5559
High	6(5.6)	5	4	
Not assessed	80(75.5)		0	
**AFP**			**0**	
Normal	48(45.3)	31	14	0.1506
High	3(2.8)	2	0	
Not assessed	55(51.9)		0	
**PIVKA-II**				
Normal	18(17.0)	14	6	0.6332
Increased	12(11.3)	9	3	
Not assessed	76(71.7)			
**SCCAg**				
Normal	20(18.9)	14	14	0.6109
Increased	8(7.5)	6	9	
Not assessed	78(73.6)			
**Prognostic group**			**0**	
Favorable	31(29.2)	19	20	0.191
Non-favorable	75(70.8)	48	11	
**Prognostic group (by Culine)**				
Good risk	64(60.4)	36	19	**0.0005**
Poor risk	42(39.6)	31	7	
**Metastasis confined to lymph node**				
Yes	36(34.0)	16	9	**0.0015**
No	70(66.0)	51	78	
**Lower GI profile**				
Lower GI profile	6(5.7)	5	13	0.126
Non-Lower GI profile	81(76.4)	54	1	
Not assessed	19(17.9)			

We assessed immunohistochemical results of CK20, CK7 and CDX-2 using TMA and analyzed expression profiles related with patients’ overall survival. In lower gastrointestinal profiles, patients with CK20 positive CUP had a poorer overall survival than patients with CK20 negative CUP (*P* = 0.002) and CDX-2 expression (*P* = 0.042). Patients with CK7 positive CUP also tended to have a shorter overall survival than patients with CK7 negative CUP (*P* = 0.111).

Patients belonging to favorable prognostic group showed better overall survival than unfavorable group with limited statistical significance (*P* = 0.191). In validation of Culine’s prognostic model, median survival of good risk patient was 19 months whereas that of poor risk patients were 7 months (hazard ratio [HR], 2.45; 95% CI, 1.46 - 4.10; *P* < 0.001).

### Multivariable analysis of clinicopathologic factors related to the patient’s survival

For adjustment of parameters affecting a patient’s survival, multivariable analysis was performed using Cox regression test (Table 3). Multivariable analysis using adenocarcinoma histology, CK20 positivity, CA19-9 elevation, metastasis confined to lymph nodes, presence of bone metastasis and favorable group defined by Culine’s prognostic model was developed. Finally, factors related with overall survival were cases belonged to Culine’s poor risk group (HR, 3.88; 95% CI, 1.75-8.64; *P* = 0.001) and CK20 positivity (HR, 3.31; 95% CI, 1.42-7.70; *P* = 0.005).

**Table 3:** Multivariable analysis of clinicopathologic factors related to survival

Variable	HR	95% CI	P
*Culine’s prognostic model*			
Poor risk group *vs*. good risk group	3.88	1.75-8.64	0.001
*Histologic subtype*			
Adenocarcinoma *vs*. other subtypes	1.51	0.76-2.97	0.24
*Lymph node metastasis*			
Lymph node involement only *vs*. lymph node and distant organ metastasis	0.86	0.34-2.16	0.76
*Bone metastsis*			
Present *vs*. absent	1.43	0.67-3.04	0.36
*CA19-9 level*			
Elevation *vs*. normal range	1.98	0.99-3.97	0.053
*CK20 expression*			
Positivity *vs*. negativity	3.31	1.42-7.70	0.005

## DISCUSSION

The clinical presentation of CUP that showed early and usually aggressive metastatic dissemination with limited life expectancy require the identification of favorable patient groups. In the present study, a consecutive series of cases of CUPs in one institute was analyzed to identify the clinicopathologic factors related to patient survival. Limited information was provided by previous literature regarding the natural course of the disease and the biologic mechanism that explain the clinical manifestation of CUP. In the early nineties, a large series with 657 consecutive patients, reported decreased survival in cases with men, more organ site, adenocarcinoma histology, metastasis to lung, bone and pleura [14]. Since then, the importance of biomarkers and their identification by IHC has been extensively researched. We validated previously reported clinicopathologic factors associated with worse prognosis such as adenocarcinoma histology, multiple metastases beyond lymph nodes, bone metastases and good risk group based on Culine’s prognostic model in this cohort. Several clinicopathologic factors related with adverse clinical outcome of CUPs were suggested including adenocarcinoma histology, poor performance status, multiple disseminated metastases, and elevated LDH, compatible with the results of this study, and they are summarized in Table 4.

**Table 4:** Previous studies of prognostic factors in patients with CUP

Reference	Number of Patients	Adverse prognostic factors	
		Univariated analysis	Multivariable analysis
Kambhu [21]	57	Poor performance status	Visceral metastases below the diaphragm
		Visceral metastases below the diaphragm	
Hainsworth [14]	220	Dominant tumor location outside retroperitoneum and peripheral lymph nodes	Dominant tumor location outside retroperitoneum and peripheral lymph nodes
		Number of metastatic sites (>3)	Number of metastatic sites (>3)
		Elevated serum CEA	Positive smoking history
		Elevated serum LDH	Older age
		Positive smoking history	
Abbruzzese [22]	657	Male	Male
		Adenocarcinoma histology	Adenocarcinoma histology
		Number of metastatic sites	Number of metastatic sites
		Lung metastases	Liver metastases
		Liver metastases	
		Bone mtastases	
		Pleura metastases	
		Brain metastases	
van der Gaast [23]	79	Poor performance status	Poor performance status
		Adenocarcinoma histology	Elevated serum alkaline phosphatase
		Bone mtastases	
		Liver metastases	
		Elevated serum alkaline phosphatase	
		Serum AST	
Culine [13]	150	Performance status 2 or 3	Performance status 2 or 3
		Liver metastases	Elevated serum LDH
		Elevated serum alkaline phosephatase	
		Elevated serum LDH	
Raghav [19]	47	Number of metastatic sites >3	Number of metastatic sites >3
		Elevated LDH	Elevated LDH
		Lung metastases	Tissue of origin not tested
		First line treatment	
		Tissue of origin not tested	

Unique findings in this cohort were the relation between expressions of biomarkers with the unfavorable clinical behaviors. CA19-9, CEA, and LDH elevation in the serum test and CK20 positivity in the tumor tissue were proven to be related with the shorter overall survival in univariate analysis. Especially, CK20 positivity was related to unfavorable overall survival in the multivariable analysis. Prognostic value of CK20 expression was validated in a subset of urothelial carcinomas and colorectal carcinomas [15-17]. This type of keratin, CK20, is a major cellular protein of mature enterocytes and goblet cells and specially expressed in the gastric and intestinal mucosa, suggesting that CUP with immunophenotype of enterocytes may have intrinsic aggressive biological behavior.

The potential limitation of this study is that the patient survival was not separately analyzed in the context of therapy given to each patient. Ever since new, broad spectrum antineoplastic chemotherapeutic drugs were introduced around late 1990s, combination of taxane and a platinum agent or gemcitabine and a platinum agent have been most commonly used for the treatment of the CUPs, but growing evidence showed a site-specific therapy based on the identified primary tumor type result in improved patient survival [3, 18]. In this regard, recent research showing epigenetic profile guided tumor type specific therapy showing improved overall survival compared with that in patients who received empiric therapy is astonishing [10]. Tests based on mRNA or miRNA profile are commercially available and they appear to show reproducible results. Although the practical use of these approaches need to be studied in the light of cost and feasibility in the clinical practice, identification of subgroups that may be eligible for site specific therapy seems to be the most important question for the improvement of survival of patient with CUP.

Prognostic model suggested by Culine was also validated in this cohort, suggesting that it would be appropriate to include this model in early clinical evaluation, considering its conciseness. Recently, Culine’s model was validated in 47 cancer of unknown primary origin in adolescents (CUP-AYA) [19]. Nevertheless, the clinical utility of this model need to be regarded in the context of the treatment as the current NCCN guideline suggests the site-specific treatment of CUP based on the information from the work-up as best as can be done.

In conclusion, clinicopathologic factors related to the patient survival was analyzed, revealing CK20 expression, adenocarcinoma histology, multiple metastases beyond lymph nodes, bone metastases, poor risk groups based on Culine’s models are factors related unfavorable outcome. Significance of these factors need to be considered in the context of eligibility of patients for the site-specific therapies.

## MATERIALS AND METHODS

### Patient selection

This retrospective study was approved by the Institutional Review Board of Yonsei University Medical Center (approval number: 4-2015-0830). A total of 106 consecutive cases from 106 patients of carcinoma of unknown primary origin were collected from the archives of pathology in Severance hospital during the period of 2000 to 2015. Patient records/information were anonymized and de-identified prior to clinicopathologic analyses. Vital status of the patient was confirmed by the national tumor registry and electronic medical record of the patients.

The diagnostic inclusion criteria were adopted from the research of previously reported literatures [1, 20].

Clinically metastatic disseminated tumor

No definite increased specific serum tumor marker that can explain the lesion.

No identified primary tumor site at presentation

Limited to epithelial and undifferentiated cancers

### Stratification of the patients

To categorize the cases into ‘favorable’ and ‘non-favorable’ groups, the following criteria were adopted from literatures [6].

Women with papillary adenocarcinoma of the peritoneal cavity

Women with adenocarcinoma involving the axillary lymph nodes

Poorly differentiated carcinoma with midline distribution

Poorly differentiated neuroendocrine carcinoma

Squamous-cell carcinoma involving cervical lymph nodes

Adenocarcinoma with a colon-cancer profile (CK20+, CK7-, CDX2+)

Men with blastic bone metastases and elevated prostate-specific antigen (adenocarcinoma)

Isolated inguinal adenopathy (squamous carcinoma)

Patients with one small, potentially resectable tumour

In addition, Culine’s prognostic model was applied and we classified the patients into group with good risk (ECOG performance status of 0 or 1 and normal LDH or no evidence of liver metastases if LDH is unknown) and poor risk (ECOG performance status of 2 or more or elevated LDH or presence of liver metastases if LDH unknown) [13].

### Generation of tissue microarray

A representative area of H&E stained slides of each tumor and the corresponding spot was marked on the paraffin block. The area was punched out by the biopsy needle and a 3-mm tissue core was placed into a recipient block. More than 2 tissue cores were extracted to minimize extraction bias. Each tissue core was assigned.

### Immunohistochemistry

We used a two-step strategy of IHC to assess CUPs according NCCN Guidelines [1]. First, we identified that tumors with occult primary tissue of origin were carcinomatous tumors using the following antibodies: broad spectrum cytokeratin (CK), S-100 protein, HMB45, and CD45. Next, staining for CK7 and CK20 is used to subtype carcinomas. We also used CDX-2 antibody because a recent report presented that patients with CUPs with lower gastrointestinal profile (CDX-2+, CK20+, CK7-) may have benefit from site-specific therapy [18]. Neuroendocrine differentiation was identified using IHCs for synaptophysin, chromogranin and CD56 in cases with histology with organoid arrangement such as solid/nesting, trabecular or gyriform pattern. Information of primary antibodies is presented as Supplementary Table 1.

Formalin-fixed, paraffin-embedded tissue sections from the tissue microarray were prepared for IHC. Briefly, 5-*μ*m-thick sections were obtained with a microtome, transferred into adhesive slides, and dried at 62°C for 30 minutes. IHC was performed using an automated staining instrument (Ventana Discovery^®^ XT, Ventana Medical System, AZ, USA) according to instruction.

### Statistics

Patients and tumor characteristics including age, gender, histologic type, treatment, immunohistochemistry against CK7, CK20 and CDX2, tumor markers (CEA, CA19-9, CA 12-5, CA 15-3, SCCAg, AFP, PIVKA-II), site of metastases were summarized using frequencies and percentages. Analysis of overall survival was performed using Kaplan-Meier’s method and log-rank tests. For adjustment of parameters affecting a patient’s survival, multivariable analysis was performed using Cox regression test. We checked the proportionality assumption of each variable using stphplot function of STATA software. Statistical significance was reached when *P* < 0.05. Data were analyzed using TitleIBM Corp. Released 2013. IBM SPSS Statistics for Windows, Version 22.0. Armonk, NY: IBM Corp.23 and Stata Statistical Software: Release 14. College Station, TX: StataCorp LP.

## SUPPLEMENTARY MATERIALS FIGURE AND TABLE


