# Supplementary material for: Prognostic clinicopathologic factors in carcinoma of unknown primary origin: a study of 106 consecutive cases
Source: Oncotarget. 2017 Mar 8;8(37):62630–40. doi: 10.18632/oncotarget.16021 (PMC5617535; doi:10.18632/oncotarget.16021)
Supplement: Supplementary file 1 [file oncotarget-08-62630-s001.pdf]

Prognostic clinicopathologic factors in carcinoma of unknown primary origin: a study of 106 consecutive cases

Supplementary Material

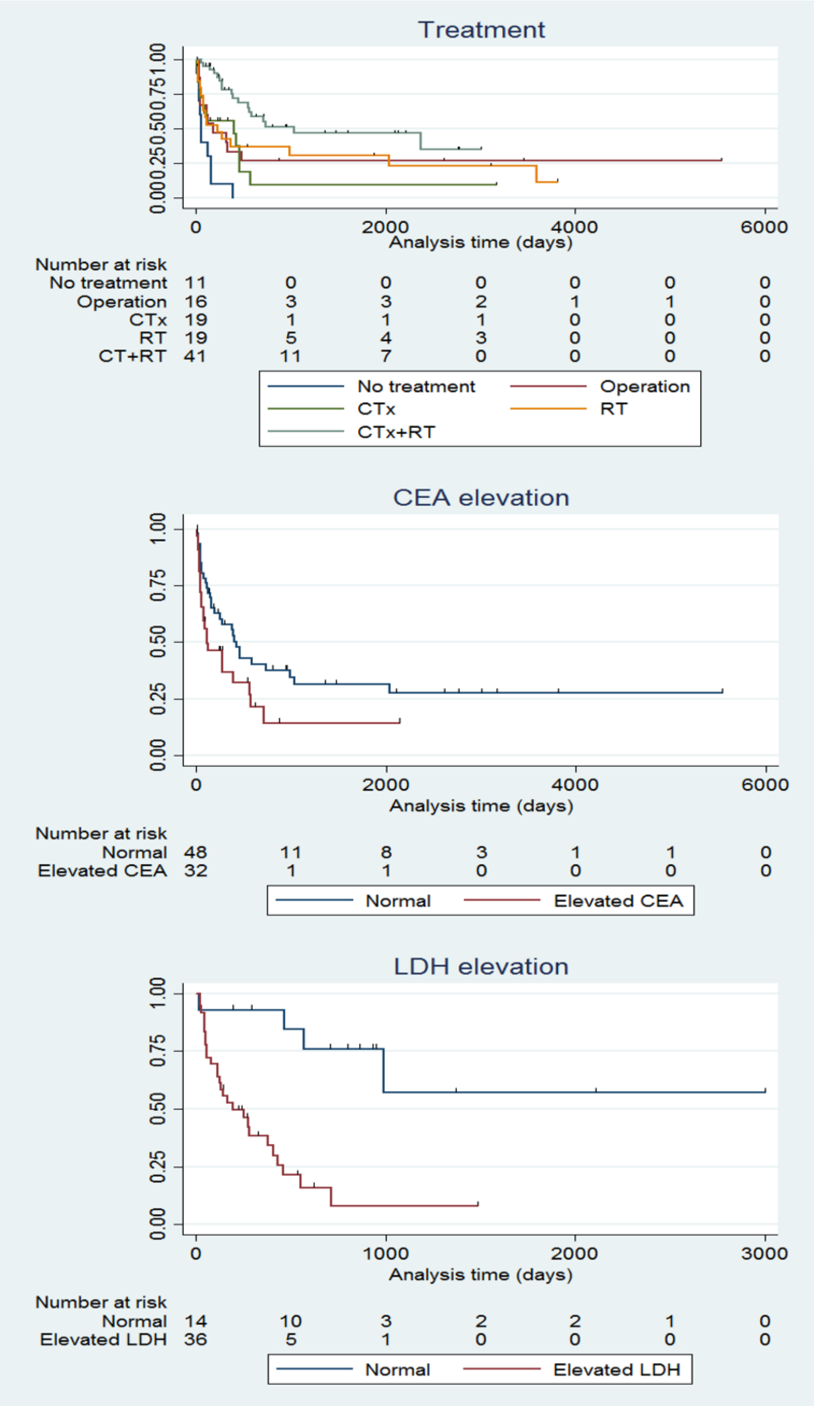

Supplementary Figure 1: Factors related with shorter overall survival was patients with no treatment (P < 0.001), increased CEA (P = 0.047), and increased lactate dehydrogenase (LDH, P < 0.001).

Supplementary Table 1.

Antibody	Industry	Catalog number	Dilution rate
CK (AE1/AE3)	DAKO	M3515	1:50
S-100 protein	DAKO	Z0311	1:400
HMB45	DAKO	M0634	1:50
CD45	DAKO	M0701	1:100
CK7	DAKO	M7018	1:100
CK20	DAKO	M7019	1:50
CDX-2	Cell marque	235R-16	1:400
Synaptophysin	DAKO	M7315	1:900
Chromogranin	DAKO	M0869	1:500
CD56	NOVOCASTRA	NCL-L-CD56-504	1:100
